# Calcitriol affects hCG gene transcription in cultured human syncytiotrophoblasts

**DOI:** 10.1186/1477-7827-6-3

**Published:** 2008-01-22

**Authors:** David Barrera, Euclides Avila, Guillermo Hernández, Isabel Méndez, Leticia González, Ali Halhali, Fernando Larrea, Angélica Morales, Lorenza Díaz

**Affiliations:** 1 Departamento de Biología de la Reproducción, Instituto Nacional de Ciencias Médicas y Nutrición Salvador Zubirán, Vasco de Quiroga No. 15, Tlalpan 14000, México, D.F; México

## Abstract

**Background:**

In pregnancy, maternal serum concentrations of calcitriol significantly rise as a result of increased renal and placental contribution in order to assure calcium supply for the developing fetus. Considering that placenta is a site for vitamin D activation, and the versatility and potency of calcitriol, it is feasible that this hormone participates in fetal/placental development and physiology. In the present work we studied calcitriol actions upon human chorionic gonadotropin (hCG) secretion and expression in cultured trophoblasts, as well as vitamin D receptor (VDR) and CYP27B1 immunolocalization in placental villi.

**Methods:**

Quantification of hCG in culture media was performed by immunoassay. Expression studies were carried out by real time PCR. Analysis of CYP27B1 and VDR localization in placental slides were performed by immunohistochemistry. Statistical significance was established by one way ANOVA using Tukey test for comparisons.

**Results:**

Calcitriol regulated hCG in a time-dependent manner: at 6 h the secosteroid stimulated hCG, whereas longer incubations (24 h) showed opposite effects. Interestingly, calcitriol stimulatory effects on hCG were accompanied by an increase in intracellular cAMP content and were abolished by pre-incubation of the cells with a selective protein kinase A inhibitor. Immunohistochemical techniques showed differential VDR localization in the syncytiotrophoblast layer or in the vascular smooth muscle cells depending on the epitope to which the antibodies were raised (specific for the carboxy- or amino-terminal regions, respectively). CYP27B1 was immunolocalized in the syncytiotrophoblast layer of placental villi.

**Conclusion:**

The presence and location of the vitamin D activating enzyme CYP27B1 as well as the specific receptor for vitamin D were shown in placental sections. The latter, together with findings demonstrating specific effects of calcitriol acting through the VDR and the cAMP/PKA signaling pathway upon hCG expression and secretion, indicate that there is a functional vitamin D endocrine system in the placenta, and recognize calcitriol as an autocrine regulator of hCG.

## Background

Vitamin D is metabolized to the steroid hormone 1,25-dihydroxyvitamin D_3 _or calcitriol, which regulates calcium homeostasis, modulates the immune response, and promotes cellular differentiation, among other actions. Calcitriol, the most active vitamin D metabolite, exerts its biological effects by binding to the vitamin D receptor (VDR), which is a ligand-activated transcription factor that recognizes cognate vitamin D response elements (VDREs) in target genes, and can also elicit rapid responses mediated by membrane receptors [1]. Placenta is a source and target of calcitriol [2]. In a similar manner to the renal process, placental production of calcitriol is catalyzed by the mitochondrial CYP27B1 [3]. In early reproductive events, calcitriol has shown to evoke specific biological effects such as regulation of the decidualization and implantation processes [4,5]. In addition, calcitriol regulates placental lactogen expression as well as progesterone and estradiol secretion in cultured human syncytiotrophoblasts [6,7]. Regarding other molecules that are regulated by calcitriol in the placenta, Evans *et al *showed that calcitriol acts in an autocrine/paracrine fashion to regulate both acquired and innate immune responses, decreasing synthesis of cytokines such as granulocyte-macrophage colony stimulating factor 2, tumor necrosis factor, and interleukin 6, but increasing expression of mRNA for the cathelicidin antimicrobial peptide [8]. Since human chorionic gonadotropin (hCG) is a pivotal hormone for pregnancy maintenance, the aim of the present work was to broaden the knowledge of calcitriol actions in the placenta, focusing in the study of its effects upon hCG expression and secretion in cultured human syncytiotrophoblasts. The data presented herein display a functional vitamin D endocrine system present in human placenta and suggest its involvement in regulating placental physiology.

## Methods

### Reagents

Culture media, fetal bovine serum (FBS) and Trizol were from Invitrogen (NY, USA). TaqMan Master reaction, TaqMan probes and the transcriptor RT system were from Roche (Roche Applied Science, IN, USA), calcitriol (1α,25-dihydroxycholecalciferol) was kindly donated from Hoffmann-La Roche Ltd (Basel, Switzerland). 3-Isobutyl-1-methylxanthine (IBMX), 8-Bromo cAMP (8-Br-cAMP), H-89 and the enzymes used for cell cultures were from Sigma-Aldrich (MO, USA). Immunoassay for hCG was from Immunometrics Ltd, (London, UK). CYP27B1 antibody (sheep anti-murine 25-hydroxyvitamin D-1α-hydroxylase) was from The Binding Site (Birmingham, UK). The VDR antibodies (rabbit polyclonal anti-VDR N-20 sc-1009 and anti-VDR C-20 sc-1008), as well as the secondary antibodies rabbit anti-sheep-horseradish peroxidase, and mouse anti-rabbit IgG-HRP were purchased from Santa Cruz Biotechnology (CA, USA). DAB (3,3'-diaminobenzidine tetrahydrochloride) was from Zymed Laboratories Inc. (CA, USA).

### Immunohistochemistry

This study was approved by the Institutional Human Ethical Committee (Hospital de Gineco-Obstetricia "Luis Castelazo Ayala", IMSS, México), and written informed consents forms were obtained from each placental donor. Term placentae (37–42 weeks of gestation) were acquired from uncomplicated pregnancies.

Fresh placental tissue from 5 term placentas was embedded in paraffin after fixation in 4% paraformaldehyde in 0.1 M phosphate buffer, pH 7.4. Serial sections (7 μm) were obtained according to standard procedures. Slides were treated with methanol-hydrogen peroxide in order to block the endogenous peroxidase activity. Normal rabbit serum and 1% BSA were used as blockers to decrease non-specific signal. Slides were then incubated with primary antibodies (anti-CYP27B1, anti-VDR-N and anti-VDR-C) during 45 minutes at room temperature, followed by further washing and incubation with secondary antibodies for another 45 minutes. Staining was developed using DAB substrate and the chromogen was contrasted with Mayer's hematoxylin. Immunolabeling specificity was tested by omitting the primary antibody.

### Trophoblast cell culture

Trophoblasts were cultured as previously described [3,9,10]. Briefly: Villous cytotrophoblasts were obtained by enzymatic dispersion and cells were separated on density Percoll gradients. Trophoblasts were plated at a density of 8 × 10^5 ^cells/mL in supplemented medium [(DMEM) 100 U/ml penicillin, 100 mg/ml streptomycin, 0.25 mg/ml Fungizone], containing 20% heat-inactivated FBS. Incubations were performed in humidified 5% CO_2_-95% air at 37°C. The morphological aspects of cells were examined daily, secreted hCG was measured by immunoassay (EIA) following manufacturer instructions and results were normalized against total protein content. Protein was determined by the method of Bradford [11].

### Calcitriol effects on hCG secretion

Two days-cultured trophoblasts were incubated in the presence of different concentrations of calcitriol or ethanol as vehicle, in serum-free DMEM-F12 during 6 h or 24 h. Additional experiments were performed incubating the cells with a selective protein kinase A inhibitor (H-89). Incubations were stopped by media collection, cell lysis with RIPA buffer (9.1 mM dibasic sodium phosphate, 1.7 mM monobasic sodium phosphate, 150 mM NaCl, 1% Nonidet P-40, 0.1% SDS, pH 7.4) was used for protein determination and hCG was quantified in culture media.

### Calcitriol effects on hCG expression

For expression studies 3 × 10^6 ^cells were plated in 25 cm^2 ^cell culture flasks and subjected to the same treatments as stated above. Total RNA was extracted using Trizol and 1 μg was reverse transcribed using the transcriptor RT system. Real-time PCR was carried out using the LightCycler 2.0 from Roche (Roche Diagnostics, Mannheim, Germany), according to the following protocol: activation of Taq DNA polymerase and DNA denaturation at 95°C for 10 min, proceeded by 45 amplification cycles consisting of 10 s at 95°C, 30 s at 60°C, and 1 s at 72°C. The primer pair was targeted to the β subunit of the hCG mRNA and the sequences were as follows: GCTCACCCCAGCATCCTAT and CAGCAGCAACAGCAGCAG. The house keeping gene glyceraldehyde-3-phosphate dehydrogenase (GAPDH) was also amplified as an internal control, using the primers: AGCCACATCGCTGAGACAC and GCCCAATACGACCAAATCC. The sizes of the resulting amplicons were 131 bp and 66 bp, and the probes utilized were # 79 and # 60 (Roche human universal probe library), for hCG and GAPDH, respectively. The expression of CYP24A1 used as a control for calcitriol effects was evaluated using the following sense and anti-sense primers: CATCATGGCCATCAAAACAA and GCAGCTCGACTGGAGTGAC and probe # 88 from Roche human universal probe library.

### Calcitriol effects on cAMP accumulation

Cells were incubated in the presence of calcitriol or its vehicle in DMEM-F12 supplemented with IBMX (0.05 mM). Incubations were terminated after 10 minutes by media collection and homogenization of the cells in RIPA buffer. Samples were boiled during 5 min for phosphodiesterases inactivation and intracellular cAMP was measured by specific radioimmunoassay (RIA) as previously described [12]. Results were normalized against total protein content and expressed as fmol cAMP/mg protein.

### Statistical analysis

Data are presented as the mean ± S.D. Statistical significance among groups was established by one way ANOVA using Tukey test. A *P *value ≤ 0.05 was considered statistically significant.

## Results

### Immunohistochemical studies

**Figure 1:**
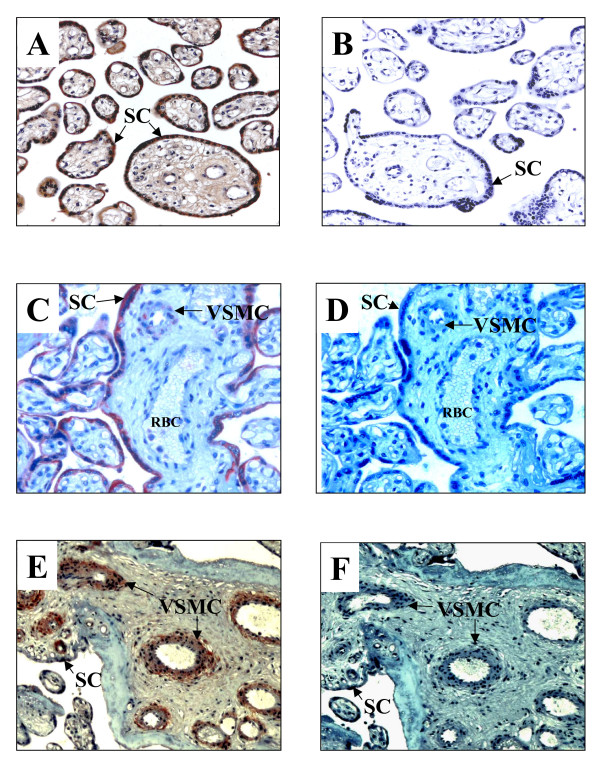
Immunolocalization of CYP27B1 and VDR in placental chorionic villi. Placental sections were incubated with specific antibodies in order to localize important components of the vitamin D endocrine system. CYP27B1 protein was located in the syncytiotrophoblast layer (A). The use of anti N-terminus VDR specific antibody disclosed intense immunostaining in the VSMC (E), whereas a VDR-C-terminus antibody preferentially stained the syncytiotrophoblast layer (C). Figure shows representative pictures of 5 different placentas. Negative controls in the absence of first antibodies are shown in B, D and F. SC = syncytiotrophoblast layer, VSMC = vascular smooth muscle cells, RBC = red blood cells. (200×).

Analysis of sequential placental tissue sections indicated the presence of immunoreactive CYP27B1 in the syncytiotrophoblast layer (Fig. 1A). A similar immunostaining pattern for VDR was visualized using an anti C-terminus specific antibody, immunostaining was also identified in vascular smooth muscle cells (VSMC, Fig. 1C). Interestingly, the use of an anti N-terminus VDR specific antibody disclosed intense immunostaining in the VSMC, and weak signal in the syncytiotrophoblast layer was observed (Fig. 1E). Control incubations without primary antibodies are shown in figure 1B, D and 1F. These results demonstrated the presence in the placenta of two important components of the vitamin D endocrine system. Considering these data, we searched for a marker of placental function. Since trophoblast cell culture has proven to be a good model to study placental physiology and hCG is a an important marker of placental functionality, we decided to use this system in order to evaluate calcitriol regulatory actions at the placental level.

### Calcitriol effects on hCG in cultured trophoblasts

**Figure 2:**
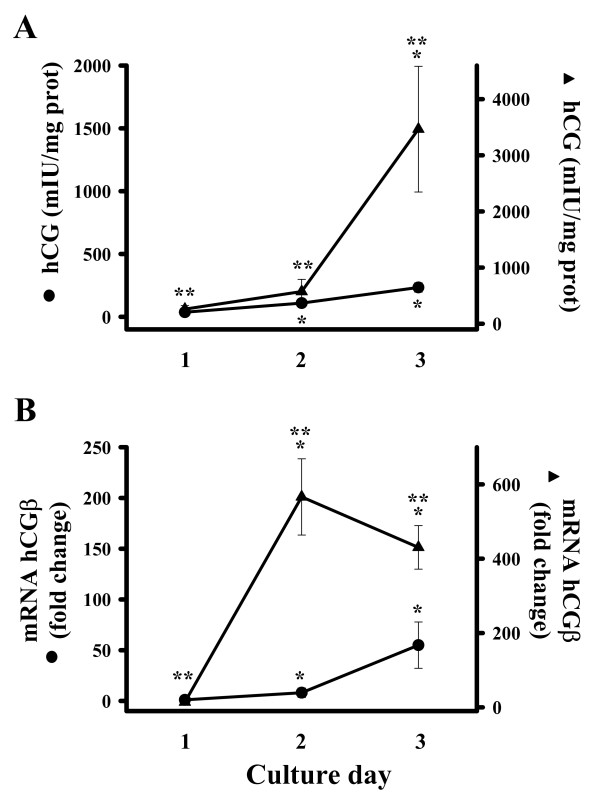
Temporal pattern of hCG secretion (A) and expression (B) in cultured human trophoblasts. Cytotrophoblasts were plated in the absence (●) or presence (▲) of 8-Br-cAMP (1.5 mM). Two scale bars were used in order to show all data [stimulated (▲) vs. non stimulated (●)] in the same graphic. Media was changed every day. A) Secretion of hCG in culture media was measured daily and results were expressed as mIU/mg protein. B) Real time PCR analysis of hCGβ expression in different culture days. Results were normalized against GAPDH mRNA. Vehicle data were arbitrarily given a value of 1. Basal hCG secretion and expression increased significantly compared with day 1. Note that hCGβ mRNA increased considerably on day 2 in the presence of 8-Br-cAMP (B), which was reflected on hCG secretion on day 3 (A), showing an important protein synthesis activity between day 2 and 3 of the cell culture. Data are presented as the mean ± S.D. of three different cell cultures. *P < 0.05 vs. day 1; **P < 0.05 vs. control.

Cultured purified cytotrophoblasts aggregated and formed syncytia in a time dependent manner. Cell culture viability was evaluated by measurements of hCG in the presence or absence of 8-Br-cAMP, an analog of cAMP, a well known regulatory factor of hCG expression [13,14]. Basal secretion of hCG into the culture media increased during the cytodifferentiation process, and cells cultured in the presence of 8-Br-cAMP secreted significantly more hCG than controls (Fig. 2A). Similarly, hCGβ mRNA increased with the same pattern observed under basal conditions (Fig. 2B), and in the presence of 8-Br-cAMP the highest hCGβ gene expression was observed on day 2, which preceded the maximal hCG secretion on day 3. These data, in addition to morphological cell evaluation, further confirmed the functional integrity of the primary culture system.

**Figure 3:**
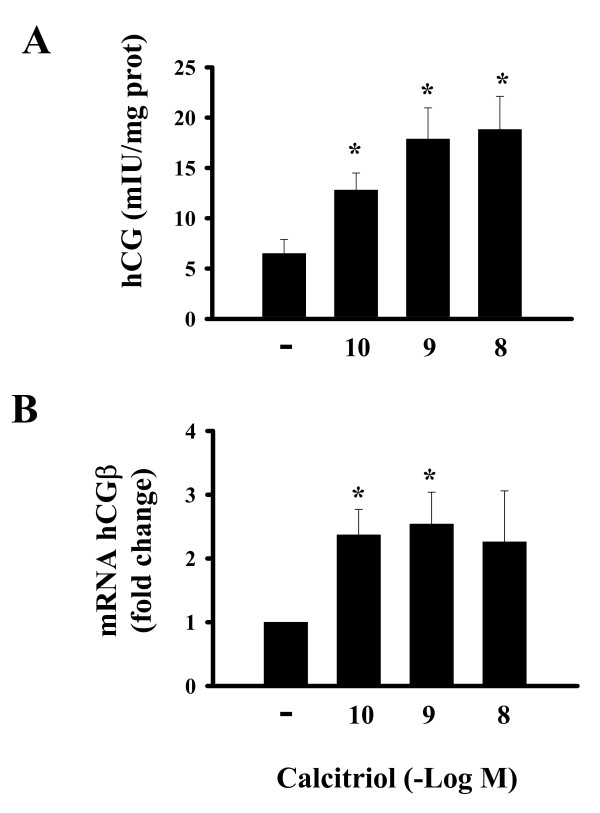
Stimulatory effects of calcitriol on hCG secretion and gene expression in cultured syncytiotrophoblasts. A) Hormone secretion was determined by EIA after 6 hours incubation in the presence of increasing concentrations of calcitriol or its vehicle (-). B) Real time PCR analysis of hCGβ gene expression of calcitriol-treated cells. Results were normalized against GAPDH mRNA. Vehicle data were arbitrarily given a value of 1. Each bar represents the mean ± S.D. of triplicate cultures. *P < 0.05 vs. control.

**Figure 4:**
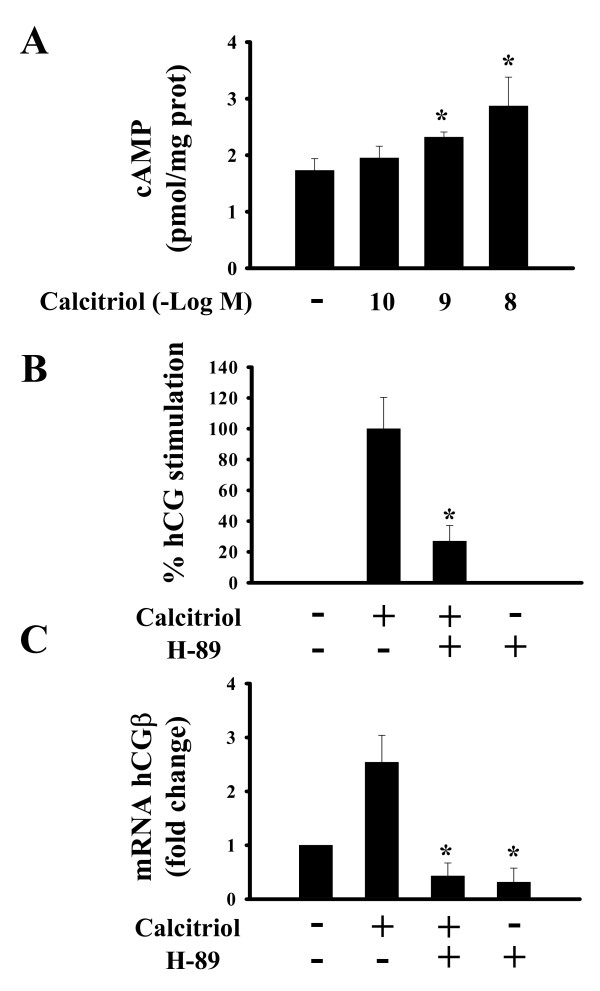
Calcitriol stimulatory effects on hCG involve cAMP. A) Dose-dependent effects of calcitriol upon intracellular cAMP. Quantification of intracellular cAMP was determined by RIA after 10 minutes incubation in the presence of increasing concentrations of calcitriol or the vehicle (-). Effects of H-89 (a selective PKA inhibitor) upon calcitriol-dependent stimulation of hCG secretion and gene expression are shown in B and C, respectively. B and C) Cells were incubated with calcitriol (1 × 10^-9 ^M) in the presence or absence of H-89 (5 μM) during 6 hours. In panel B, calcitriol incubations in the absence of H-89 represent 100% stimulation. In panel C results were normalized against GAPDH mRNA, giving vehicle data an arbitrarily value of 1. Each bar represents the mean ± S.D. of triplicate experiments. *P < 0.05 vs. control.

In the presence of calcitriol, hCG secretion increased significantly after 6 hours of incubation (Fig. 3A). Calcitriol also upregulated hCGβ mRNA (Fig. 3B). Since hCG is highly regulated by the cAMP/PKA pathway, and calcitriol has shown to induce cAMP accumulation [12,15], we investigated the participation of this second messenger upon hCG-upregulation by calcitriol. For this purpose, cAMP was quantified in calcitriol-incubated cells. Results indicated that after 10 minutes of treatment, the secosteroid significantly increased intracellular cAMP content in a dose-dependent manner (Fig. 4A). Preincubation of cells with a selective inhibitor of PKA (H-89) reduced hCG expression below basal levels and prevented the calcitriol-dependent protein and gene hCG-stimulation detected at the 6 h incubation period (Fig. 4B and 4C, respectively). The same results were obtained in 72 h cultures (data not shown).

**Figure 5:**
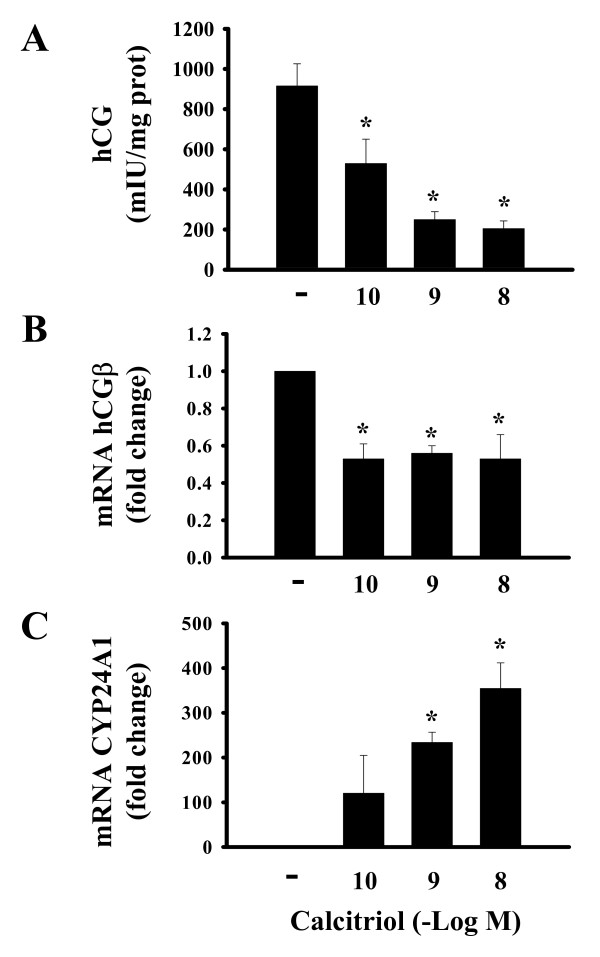
Long-term inhibitory effects of calcitriol on placental hCG. A) hCG concentration in culture medium was determined after 2 days incubation in the presence of increasing concentrations of calcitriol or the vehicle alone (-). B) Real time PCR analysis of hCGβ gene expression in calcitriol-treated cells after 24 h. Results were normalized against GAPDH mRNA, vehicle data were arbitrarily given a value of 1. C) CYP24A1 mRNA induction by calcitriol after 24 h. Results were normalized against GAPDH mRNA. Vehicle data were arbitrarily given a value of 1. The inhibitory effects of calcitriol upon hCG gene expression detected after 24 h (B) were reflected in a reduction of secreted hCG after 48 h (A). These effects were not due to a toxic effect of calcitriol since cells showed increased CYP24A1 gene expression, as expected (C). Each bar represents the mean ± S.D. of triplicate experiments. *P < 0.05 vs. control.

Calcitriol long-term effects upon hCG were also studied. The stimulatory effects observed at 6 h were no further evident after 24 h (data not shown), and when cells were incubated in the presence of calcitriol during 2 consecutive days, the effects were rather inhibitory (Fig. 5A). Inhibition was evident at the mRNA level after 24 hours treatment (Fig. 5B), preceding the observed response in hCG protein. This repressive calcitriol effect could not be attributed to decreased cell viability, since under the same conditions, calcitriol upregulated CYP24A1 gene expression (Fig. 5C).

## Discussion

Serum concentrations of biologically active hCG depend on the rate of synthesis of its specific β subunit; whereas at the cellular level multiple factors modulate hCG production by interacting with specific membrane receptors on placental trophoblasts. The most studied factors that modulate hCG are the gonadotropin releasing hormone (GnRH), hCG itself and other molecules that activate cAMP-dependent signal transduction pathways [16-18]. Indeed, both hCGα and hCGβ genes are highly transcriptionally induced by cAMP [19-21]. In this study we showed that calcitriol is an additional factor that modulates hCG in human trophoblasts. In fact, calcitriol regulated hCG in a time-dependent manner, stimulating or inhibiting hormone secretion and expression. The stimulatory effect after 6 hours was probably due, as demonstrated in this study, to the rapid calcitriol-dependent increase in intracellular cAMP. This assumption was further supported by results in the presence of H-89, a selective inhibitor of PKA. Indeed, blocking the cAMP/PKA signal transduction cascade impaired the ability of calcitriol to elicit transcriptional induction of hCGβ gene, as well as hCG secretion into the culture media. Rapid cAMP generation induced by calcitriol has been previously reported in other cell types [15,22], and may be the result of its interaction with membrane-VDR or other surface proteins. In addition, since it has been demonstrated that calcium ion channels are involved in GnRH dependent-hCG secretion [23], calcitriol could also release stored hCG through promoting a rapid calcium entry into the cell. Further studies are needed in order to clarify this matter.

The concentration of hCG was also measured after 12, 24 and 48 h of calcitriol treatment, but the results reported in the present study were only those that differed significantly when compared with the vehicle alone. The stimulatory effects observed at 6 h were no further evident after 12 or 24 h, and when cells were incubated in the presence of calcitriol during 2 consecutive days, the effects were rather inhibitory. Inhibition was evident at the mRNA level after 24 hours treatment, preceding the observed response in hCG protein. These data are probably more likely to be reflective of the true biological situation. Indeed, our results that calcitriol inhibited hCG were in line with previous data from this and other laboratories where low serum calcitriol and high serum hCG levels were found in preeclampsia [24-26], that conjointly with the fact that [^3^H]25-hydroxyvitamin D bioconversion into [^3^H]1,25-dihydroxyvitamin D was significantly reduced in preeclamptic placentas [9], may suggest a direct regulatory effect of calcitriol on hCG production. Regarding the inhibitory effects of calcitriol on hCG, it is likely that a secondary metabolic C23/C24 calcitriol oxidation pathway might play a role, since the resulting trihydroxylated metabolite is considered biologically inactive [1]. Alternatively, since calcitriol has been shown to stimulate progesterone secretion [6] and in turn this hormone inhibits hCG secretion [27], this mechanism could additionally participate in calcitriol long term inhibitory effects in placenta. In any case, the demonstration in this study of genomic mediated effects of calcitriol on hCG suggested the presence of VDR dependent regulatory regions on hCG promoters. Indeed we found five putative VDR/RXR heterodimer binding sites in the hCGβ-5 gene promoter [28], which probably may be acting as calcitriol dependent-transcriptional regulatory regions. Nevertheless, the sole presence of the VDREs in the hCGβ-5 promoter is not sufficient to indicate transcriptional function; therefore, functional evaluation of the putative VDREs deserves further investigation.

In non pregnant women the physiological concentration of calcitriol fluctuates between 40–100 pM. In the present study the calcitriol doses tested were: 100 pM, 1 nM and 10 nM. The lowest concentration (100 pM) is within the physiological range of circulating calcitriol levels in mexican pregnant women (127 pM and 151 pM) as observed previously [24,29]. The other doses tested were supraphysiological, nevertheless, calcitriol effects upon hCG were evident starting with the lowest concentration.

Placenta is considered not only as a source but also as a target of calcitriol [2]. In order to get insights on calcitriol paracrine/autocrine effects in placenta, we investigated the immunolocalization of VDR and CYP27B1 in placental chorionic villi. In accordance with previous reports [30], CYP27B1 protein was located in the syncytiotrophoblast layer, corroborating that the endocrine phenotype of trophoblasts cells is responsible for vitamin D activation in placenta. To answer where the locally produced calcitriol acts in the placenta, we looked for VDR protein in placental sections. To our knowledge, this is the first report to show immunoreactive VDR in different locations in the placental villi, since VDR expression has been mainly addressed at the mRNA level in placenta [2,31]. The antibodies showed the presence of VDR in the endocrine placental cells and VSMC, suggesting that calcitriol could be involved in regulating hormonal production and vascular remodeling through the VDR. The latter assumption derives from previous studies demonstrating that calcitriol acts in the vasculature promoting VSMC growth and migration [32,33]. Interestingly, the C-terminus antibody intensely stained the syncytiotrophoblast layer and faintly stained the surrounding cells of placental vessels, whereas the N-terminus antibody detected a strong signal in the endothelial and VSMC. These observations may indicate different epitopes recognized by the antibodies depending on the topological position of the VDR. An interesting challenge would be to define specific VDR responses in different placental structures.

## Conclusion

In summary, the present study broadens the knowledge of placental vitamin D endocrine system by demonstrating the physiological effects of calcitriol on an important biochemical placental function marker such as hCG. In addition, this is the first report to show immunoreactive VDR in different locations in human placental villi, and opens the field to address important research questions on the role of calcitriol in specific placental structures.

## Competing interests

The author(s) declare that they have no competing interests.

## Authors' contributions

DB carried out real time PCR's analysis, hCG quantification and participated in the design of the study and statistical analysis. EA participated in the design of the study, particularly the molecular studies, performed real time PCR analysis and helped to draft the manuscript. GH performed placenta collection, trophoblast primary cell cultures, RNA extraction and reverse transcription reactions. IM and LG were in charge of all experiments concerning cAMP, including design and analysis of the results. AH contributed in interpretation of data and was involved in drafting the manuscript. FL made substantial contribution to the design of the study, was involved in drafting the manuscript and revised it critically. AM performed the immunohistochemical studies and helped to draft the manuscript. LD conceived the study, participated in the design and coordination, structured the manuscript and actively participated in experimental procedures. All authors read and approved the final manuscript.
